# Supplementary material for: The Need for Red Cell Support During Non-Cardiac Surgery Is Associated to Pre-Transfusion Levels of FXIII and the Platelet Count
Source: J Clin Med. 2020 Jul 31;9(8):2456. doi: 10.3390/jcm9082456 (PMC7465630; doi:10.3390/jcm9082456)
Supplement: Supplementary file 1 [file jcm-09-02456-s001.pdf]

Pat._ID	Fibrinogen	FXIII	Platelet Count	RBC concentrates transfused
D00001	2.96	0.83		2
D00002	3.02	0.5	152	0
D00003	1.8	0.42	111	2
D00004	6.27	0.81	46	0
D00005	2.54	0.31	299	2
D00006	3.26	1.17	265	2
D00007	2.46	0.39	195	2
D00008	3.17	0.46	79	0
D00009	1.78	0.46	241	2
D00010	2.69	0.47	196	1
D00011	2.9	0.7	126	2
D00012	2.96	0.54	412	0
D00013	2.33	0.48	266	6
D00014	2.35	0.68	203	2
D00015	2	0.55	93	4
D00016	5.6	0.69	151	0
D00017	1.43	0.53	82	3
D00018	1.48	0.63	294	3
D00019	2.03	1.01	251	0
D00020	3.1	0.44	109	0
D00021	2.4	1.33	149	1
D00022	1.98	0.63	189	0
D00023	1.48	0.51	131	3
D00024	2.62	1.03	246	0
D00025	2.64	0.68	245	0
D00026	2.31	0.84	95	0
D00027	3.17	1.17	145	4
D00028	2.84	0.45	184	0
D00029	3.07	0.9	143	0
D00030	3.89	0.66	87	2
D00031	1.3	0.57	151	3
D00032	2.27	0.43	224	3
D00033	2.21	0.92	77	0
D00034	3.64	0.63	143	2
D00035	1.4	0.38	62	6
D00036	3.18	1.04	197	0
D00037	2.54	0.92	239	0
D00038	1.88	0.7	197	2
D00039	1.4	0.42	114	2
D00040	2.96	1.19	304	0
D00041	2.49	0.87	124	0
D00042	2.64	0.71	104	0
D00043	0.94	0.46	96	1
D00044	3.42	0.75	202	0
D00045	2.27	0.63	40	4
D00046	4.08	0.77	336	0
D00047	2.68	1.47	236	0
D00048	2.84	1.01	235	0
D00049	3.22	0.92	64	2
D00050	1.86	0.61	138	0
D00051	4.64	0.81	245	2
D00052	2.23	0.81	273	0
D00053	2.84	0.7	115	0
D00054	1.72	0.66	332	0
D00055	2.62	1.12	257	0
D00056	2	0.72	140	2

D00057	0.86	0.54	112	1
D00058	1.82	0.33	304	0
D00059	2.49	0.53	259	4
D00060	6.03	0.8	313	0
D00061	1.69	0.78	194	2
D00062	2.49	0.82	119	0
D00063	3.59	1.17	220	0
D00064	2.7	0.58	138	3
D00065	1.2	0.5	119	6
D00066	2.69	1.39	133	2
D00067	2.03	0.99	146	0
D00068	1.51	0.58	227	2
D00069	3	0.93	153	2
D00070	2.42	0.61	162	0
D00071	2.27	0.67	221	0
D00072	2.49	0.91	230	0
D00073	2.21	1.31	217	0
D00074	2.03	0.56	118	2
D00075	1.92	0.39	118	1
D00076	2.51	0.39	36	2
D00077	2.33	0.85	63	0
D00078	2.15	1.18	193	0
D00079	4.95	0.53	1042	0
D00080	1.83	0.56	75	7
D00081	1.39	0.59	156	0
D00082	2.78	0.4	167	0
D00083	2.15	0.57	62	0
D00084	2.81	0.91	203	0
D00085	2.56	0.75	84	1
D00086	1.46	1.04	79	0
D00087	3.25	1.09	242	0
D00088	2.1	1.17	148	2
D00089	2.73	0.97	217	0
D00090	2.56	0.92	129	0
D00091	2.15	0.78	235	0
D00092	1.77	0.95	101	1
D00093	1.12	0.7	121	0
D00094	2.44	1.23	123	0
D00095	0.4	0.1	66	4
D00096	5.4	0.43	270	4
D00097	1.83	0.96	98	3
D00098	2.4	0.59	117	0
D00099	2.03	0.5	184	0
D00100	1.52	0.45	110	2
D00101	3.07	0.95	147	0
D00102	2.15	0.87	209	0
D00103	2.14	0.47	285	2
D00104	2.25	1.18	108	4
D00105	2.78	1.16	225	0
D00106	1.53	0.47	207	1
D00107	3.22	1.09	151	3
D00108	2.91	0.67	163	1
D00109	4.26	1.7	323	0
D00110	3.59	0.63	280	0
D00111	2.37	0.29	47	0
D00112	7.1	0.45	141	3
D00113	5.21	0.9	141	4
D00114	1.73	0.36	189	3
D00115	1.97	0.76	108	0

D00116	2.87	1.2	140	0
D00117	2.03	0.74	92	0
D00118	1.6	0.91	61	0
D00119	1.78	0.56	155	2
D00120	1.58	0.5	79	0
D00121	3.43	0.95	215	0
D00122	3.22	1.13	175	0
D00123	4.68	0.4	395	2
D00124	5.6	0.42	166	4
D00125	2.06	1.26	91	0
D00126	1.79	0.46	112	0
D00127	3.18	1.38	191	0
D00128	2.31	1.12	150	3
D00129	2.25	0.76	110	0
D00130	2.35	0.55	175	0
D00131	0.1	0.37	41	3
D00132	1.67	0.24	249	2
D00133	3.38	1.16	227	0
D00134	2.42	0.57	158	3
D00135	2.01	0.46	129	0
D00136	1.82	0.65	155	3
D00137	1.21	0.63	80	2
D00138	4.57	0.88	250	0
D00139	2	0.19	102	0
D00140	1	0.96	129	0
D00141	1.57	0.81	300	0
D00142	2.25	0.98	153	0
D00143	2.49	0.5	174	0
D00144	3.47	0.59	278	0
D00145	1.09	0.61	112	2
D00146	4.2	1.69	197	0
D00147	0.94	0.37	128	2
D00148	2.9	1.31	110	3
D00149	1.81	0.94	187	0
D00150	1.86	0.75	230	0
D00151	3.84	0.69	261	3
D00152	3.73	0.53	440	0
D00153	3.41	0.28	100	0
D00154	2.13	0.98	239	0
D00155	5.55	0.31	101	0
D00156	2.87	1.14	157	0
D00157	2.94	0.87	95	0
D00158	2.37	1.09	78	0
D00159	1.54	1.17	80	0
D00160	1.69	0.95	69	0
D00161	0.88	0.47	62	4
D00162	1.41	0.89	181	0
D00163	2.87	0.83	153	4
D00164	4.79	0.77	177	0
D00165	2.21	0.76	257	0
D00166	4.3	0.45	169	0
D00167	2.67	0.57	105	0
D00168	7.92	0.56	314	0
D00169	1.81	0.27	54	1
D00170	3.51	1.1	241	0
D00171	2.1	0.84	156	0
D00172	2.35	0.69	166	2
D00173	2.64	0.59	172	0
D00174	1.67	0.51	115	0

D00175	1.76	0.48	252	2
D00176	1.67	0.36	122	4
D00177	2.42	0.99	218	0
D00178	1.71	0.65	102	0
D00179	5.12	0.95	398	0
D00180	4.2	0.82	436	3
D00181	3.17	0.85	125	0
D00182	5.76	0.54	308	0
D00183	0.7	0.57	148	0
D00184	2.19	1	28	10
D00185	3.75	1.07	192	0
D00186	4.91	1.39	187	0
D00187	2.29	1.04	232	0
D00188	7.81	0.71	99	0
D00189	1.58	0.38	88	0
D00190	2.73	1.04	252	0
D00191	2.59	0.85	117	0
D00192	1.79	0.49	157	2
D00193	3.54	0.73	174	0
D00194	2.25	0.48	149	1
D00195	1.1	0.27	113	2
D00196	1.88	0.61	78	4
D00197	2.23	0.88	98	0
D00198	1.58	0.75	209	0
D00199	5.08	0.37	192	0
D00200	4.24	0.27	169	2
D00201	2.42	0.67	41	0
D00202	1.18	0.45	55	2
D00203	1.73	0.58	110	0
D00204	1.12	0.23	119	7
D00205	2.35	0.6	110	0
D00206	1.54	0.45	93	0
D00207	1.88	0.35	237	5
D00208	1.92	0.77	9	5
D00209	1.98	0.58	98	0
D00210	6.67	0.56	52	4
D00211	1.86	0.7	141	2
D00212	4.24	0.33	243	2
D00213	2.87	0.5	112	2
D00214	2.59	0.94	124	0
D00215	3.25	0.91	165	0
D00216	2.15	0.72	155	0
D00217	4.87	0.73	163	2
D00218	5.04	0.88	257	4
D00219	2.64	1.03	43	0
D00220	2	0.75	376	0
D00221	2.25	0.84	277	0
D00222	4.24	0.62	407	2
D00223	3.84	0.42	174	3
D00224	2.56	0.99	255	0
D00225	1.67	0.74	168	0
D00226	2.47	0.55	179	0
D00227	1.39	0.53	44	6
D00228	3.21	0.8	162	0
D00229	2.75	1.28	122	0
D00230	2.29	0.83	285	0
D00231	0.66	0.27	45	2
D00232	2.27	0.77	169	0
D00233	2.25	1.47	224	0

D00234	2.31	0.45	61	10
D00235	1.03	0.32	122	9
D00236	3.6	1.29	156	0
D00237	2.78	0.67	159	0
D00238	2.96	0.51	236	1
D00239	2.03	0.72	147	5
D00240	1.91	0.66	182	4
D00241	2.1	0.95	90	0
D00242	2.35	0.75	216	0
D00243	1.91	0.86	126	0
D00244	2.21	0.52	135	0
D00245	1.91	0.87	104	4
D00246	2.51	1.15	245	0
D00247	3.7	1.07	155	0
D00248	2.29	1.01	296	0
D00249	2.19	0.4	101	3
D00250	2.67	0.52	80	2
D00251	1.49	0.29	118	0
D00252	2.25	0.51	196	0
D00253	5.4	1.28	236	0
D00254	5.7	0.89	593	0
D00255	2.49	0.59	105	0
D00256	4.43	0.37	195	1
D00257	2	0.53	54	0
D00258	3.41	0.77	94	0
D00259	2.51	0.91	390	0
D00260	2.49	1.11	374	0
D00261	5.49	0.64	266	0
D00262	2.01	0.67	215	2
D00263	1.74	0.48	364	3
D00264	2.47	0.95	154	0
D00265	2.33	0.84	194	0
D00266	4.64	0.66	185	0
D00267	2	0.73	121	0
D00268	5.35	0.41	179	3
D00269	1.68	0.48	117	0
D00270	3.3	0.85	136	0
D00271	1.89	0.86	179	0
D00272	2.05	0.85	112	1
D00273	2.29	0.49	94	2
D00274	1.6	0.57	200	0
D00275	4.87	1.25	275	0
D00276	2	0.75	48	8
D00277	7.49	0.66	191	0
D00278	2.59	0.82	53	4
D00279	2.78	0.54	110	0
D00280	2	0.66	81	0
D00281	1.59	0.61	128	0
D00282	2.03	0.74	98	0
D00283	2.97	0.81	123	0
D00284	2.17	0.53	119	3
D00285	3.3	1.15	291	0
D00286	2.19	1.15	233	0
D00287	2.4	0.96	171	0
D00288	1.58	0.8	70	4
D00289	1.78	1.01	169	4
D00290	1.76	0.61	96	4
D00291	2.12	0.39	156	6
D00292	0.65	0.18	54	4

D00293	1.86	0.79	226	2
D00294	4.6	0.5	421	0
D00295	2.97	1.04	196	0
D00296	1.72	0.73	121	2
D00297	3.07	1.11	261	2
D00298	2.33	1	225	0
D00299	2.15	0.9	86	0
D00300	1.82	1.14	111	3
D00301	2.78	1.32	381	0
D00302	2.4	0.52	16	4
D00303	2.81	0.91	216	0
D00304	3.29	0.79	134	0
D00305	5.21	0.74	391	2
D00306	4.32	0.91	17	4
D00307	6.03	0.32	243	2
D00308	4.32	0.91	17	0
D00309	1.48	0.36	109	10
D00310	4.83	1.34	311	0
D00311	2.23	0.79	133	2
D00312	1.09	0.56	68	0
D00313	4.5	0.8	180	0
D00314	4.46	0.99	29	1
D00315	2.59	0.36	70	2
D00316	3.91	1.17	310	0
D00317	4.99	0.92	716	2
D00318	1.61	1.05	49	0
D00319	1.78	0.65	172	2
D00320	1.78	0.43	127	0
D00321	6.03	1.11	330	0
D00322	0.59	0.96	92	0
D00323	1.69	0.52	383	0
D00324	1.6	0.41	220	2
D00325	2.75	0.51	160	0
D00326	2.17	0.81	152	0
D00327	2.1	0.38	74	3
D00328	1.92	0.59	55	2
D00329	1.64	0.57	167	4
D00330	2.73	1.05	251	0
D00331	1.42	0.51	142	0
D00332	2.61	0.85	341	0
D00333	2.51	0.71	142	0
D00334	3.54	0.6	492	0
D00335	6.27	0.64	243	2
D00336	2.15	0.79	174	2
D00337	3.42	1.22	200	0
D00338	0.91	0.23	62	4
D00339	3.18	0.65	87	3
D00340	2.15	0.78	187	0
D00341	2.25	0.55	150	0
D00342	1.6	0.71	187	0
D00343	3.25	0.45	250	0
D00344	1.48	0.4	125	2
D00345	4.75	0.54	79	0
D00346	1.08	0.62	113	0
D00347	1.97	0.38	159	2
D00348	1.82	0.45	108	0
D00349	1.78	0.37	102	0
D00350	1.42	0.7	156	0
D00351	1.05	0.87	221	0

D00352	4	0.57	55	6
D00353	1.95	0.76	81	8
D00354	1.72	0.37	299	4
D00355	2.61	1	243	0
D00356	5.17	0.69	17	0
D00357	2.78	0.33	511	4
D00358	2.59	0.84	165	0
D00359	1.76	0.67	157	0
D00360	2.44	0.53	134	0
D00361	2.23	0.76	133	2
D00362	1.74	0.63	110	0
D00363	2.06	0.47	158	6
D00364	6.03	0.72	137	2
D00365	2.19	0.96	120	0
D00366	1.54	0.67	4	0
D00367	1.37	0.59	71	0
D00368	2.4	0.57	286	0
D00369	2.19	0.56	105	4
D00370	1.58	0.74	189	0
D00371	3.17	0.74	67	6
D00372	2.73	0.95	310	0
D00373	2.94	1	291	0
D00374	2.9	0.99	175	0
D00375	2.94	0.53	170	0
D00376	2.9	0.75	73	0
D00377	2.78	0.19	346	2
D00378	2.64	1.01	226	0
D00379	1.97	0.82	165	0
D00380	1.52	0.55	126	10
D00381	2.01	0.82	172	0
D00382	1.31	0.78	187	0
D00383	1.08	0.29	164	6
D00384	2.15	1.2	201	0
D00385	3.47	0.93	200	0
D00386	3.29	0.57	141	3
D00387	1.6	0.52	163	0
D00388	3.25	0.91	186	0
D00389	1.07	0.31	42	5
D00390	2.64	0.65	206	3
D00391	6.67	1.08	410	0
D00392	0.94	0.37	135	3
D00393	1.22	0.49	58	1
D00394	5.04	0.4	306	3
D00395	2.37	0.21	494	3
D00396	3.3	0.76	234	0
D00397	2.96	0.5	76	4
D00398	2.35	0.77	137	0
D00399	1.61	0.4	57	5
D00400	3.68	1.08	395	1
D00401	2.23	0.49	108	4
D00402	1.83	0.34	202	1
D00403	4.79	0.58	222	0
D00404	1	0.4	133	0
D00405	1.07	0.5	43	0
D00406	4.79	0.64	720	3
D00407	5.21	0.4	74	4
D00408	2.75	0.9	375	0
D00409	1.92	0.73	138	0
D00410	4.46	0.73	25	0

D00411	1.81	0.73	22	0
D00412	2.9	0.43	60	4
D00413	4.06	0.86	231	0
D00414	5.81	0.74	197	2
D00415	0.4	0.49	73	2
D00416	3.07	0.83	189	0
D00417	0.84	0.49	128	3
D00418	2.59	1	169	0
D00419	2.64	0.53	406	2
D00420	2.27	0.63	231	3
D00421	5.17	0.86	194	0
D00422	3.17	0.87	322	0
D00423	2.38	0.81	204	0
D00424	1.71	0.76	137	0
D00425	2.56	0.57	182	0
D00426	1.82	0.78	109	0
D00427	2.59	0.53	155	2
D00428	3.41	1.51	599	0
D00429	2.33	1.13	112	7
D00430	2.73	1.07	307	0
D00431	3.07	1.29	134	0
D00432	2.61	0.79	139	0
D00433	2.35	0.61	23	1
D00434	1.48	0.9	87	2
D00435	3.18	1.16	249	0
D00436	4.32	1.5	174	0
D00437	2.73	1.56	227	0
D00438	1.88	0.45	131	0
D00439	1.98	0.65	154	4
D00440	2.08	1.09	112	4
D00441	1.85	0.98	71	0
D00442	1.29	0.64	72	0
D00443	2.19	0.96	175	0
D00444	2.21	0.45	63	10
D00445	1.68	0.98	174	0
D00446	1.56	0.84	98	0
D00447	2.49	1.56	135	15
D00448	2.33	0.59	215	2
D00449	1.73	0.61	125	0
D00450	3.73	0.99	300	0
D00451	2.13	0.46	115	3
D00452	2.42	1.19	150	0
D00453	1.91	1.14	233	0
D00454	1.98	1.47	103	4
D00455	2.84	1.31	294	0
D00456	6.67	0.9	272	0
D00457	1.57	0.52	63	0
D00458	1.56	0.58	102	0
D00459	2.33	0.29	60	4
D00460	5.21	0.64	185	0
D00461	2.35	0.72	180	0
D00462	2.37	0.9	111	2
D00463	5.81	0.41	56	2
D00464	1.72	0.88	147	0
D00465	4.36	0.46	234	0
D00466	1.82	1.01	59	6
D00467	0.8	0.46	82	4
D00468	4.06	0.82	240	0
D00469	2.91	1.53	330	0

D00470	2.59	0.49	325	2
D00471	2.47	0.97	188	0
D00472	3.17	0.58	289	0
D00473	0.8	0.63	47	0
D00474	6.03	1.07	8	0
D00475	5.81	0.82	9	0
D00476	2.21	0.98	131	0
D00477	5.08	0.48	81	1
D00478	4.11	1.3	252	0
D00479	4.14	0.4	104	2
D00480	2.06	0.6	225	0
D00481	1.77	0.38	143	0
D00482	1.18	0.34	98	2
D00483	2.08	0.56	128	9
D00484	5.45	0.71	264	2
D00485	2.23	0.78	166	0
D00486	2.21	0.52	151	2
D00487	5.4	0.51	191	2
D00488	6.11	0.54	177	0
D00489	2.06	0.78	206	6
D00490	3.51	0.48	307	2
D00491	3.18	1.42	267	0
D00492	3.42	0.6	398	0
D00493	5.04	0.45	118	4
D00494	0.85	0.25	216	2
D00495	1.41	0.51	190	0
D00496	4.95	0.74	14	0
D00497	2.03	1.23	157	0
D00498	5.4	0.35	223	2
D00499	4.64	1.03	306	0
D00500	2.56	0.55	308	0
D00501	2.05	0.77	92	3
D00502	1.29	0.53	68	4
D00503	3.21	1.39	113	8
D00504	2.35	0.68	176	0
D00505	6.27	1.55	340	0
D00506	4.11	0.49	209	4
D00507	1.98	0.49	120	0
D00508	1.86	0.5	110	5
D00509	4.53	1.13	337	0
D00510	5.3	1.07	6	0
D00511	2.03	0.56	52	0
D00512	3.16	1.32	142	2
D00513	3.1	0.48	154	4
D00514	1.86	0.32	257	3
D00515	2.72	0.43	126	6
D00516	1.98	0.47	161	1
D00517	2.87	0.79	327	0
D00518	1.71	0.25	152	4
D00519	2.31	0.64	174	2
D00520	1.71	0.38	100	4
D00521	2.54	0.83	224	0
D00522	2.15	0.89	197	0
D00523	4.19	1.43	218	0
D00524	1.41	0.47	118	0
D00525	5.92	0.85	113	0
D00526	5.47	0.31	212	3
D00527	2.25	1.2	72	8
D00528	2.61	0.92	507	2

D00529	1.41	0.63	174	0
D00530	4.85	1	396	0
D00531	1.67	0.49	160	0
D00532	1.71	0.65	180	0
D00533	1.43	0.25	120	10
D00534	5.55	0.85	432	0
D00535	1.78	1.16	117	6
D00536	4.77	0.83	175	0
D00537	4.86	0.7	6	0
D00538	2.01	0.23	57	6
D00539	2.66	0.9	147	0
D00540	2.58	0.46	106	0
D00541	2.08	0.42	100	0
D00542	1.84	1.09	203	0
D00543	2.39	0.92	129	0
D00544	0.9	0.18	212	0
D00545	2.71	0.46	210	2
D00546	3.36	1.42	200	0
D00547	1.8	0.55	113	0
D00548	4.57	1.1	173	0
D00549	2.05	0.72	138	0
D00550	1.87	1.04	110	0
D00551	4.14	0.84	582	5
D00552	1.96	0.36	263	0
D00553	2.07	0.79	115	0
D00554	3.44	1.2	184	0
D00555	2.79	0.99	145	0
D00556	4.13	1.53	181	0
D00557	1.89	0.75	132	0
D00558	2.24	0.52	171	0
D00559	2.18	0.59	273	0
D00560	5.41	0.94	203	0
D00561	2.17	0.76	164	0
D00562	0.8	0.57	78	2
D00563	2.35	0.98	100	0
D00564	2.47	0.85	178	0
D00565	1.58	0.49	78	2
D00566	3.22	0.66	141	0
D00567	0.8	0.36	43	2
D00568	3.33	1.34	163	0
D00569	2.29	0.37	164	0
D00570	3.41	0.81	11	0
D00571	4.26	1.14	8	0
D00572	3.05	0.55	366	0
D00573	1.53	0.88	109	2
D00574	6.85	0.88	12	0
D00575	3.56	0.63	209	2
D00576	1.91	0.89	55	12
D00577	6.32	1.31	10	0
D00578	3.83	0.49	365	0
D00579	2.58	0.54	94	0
D00580	2.71	1.25	72	3
D00581	4.71	0.78	9	0
D00582	2.05	0.85	183	0
D00583	1.48	0.61	127	2
D00584	1.38	0.64	44	12
D00585	3.45	0.66	19	0
D00586	1.91	0.65	127	2
D00587	2.99	0.3	108	3

D00588	2.9	0.54	188	3
D00589	3.07	1.67	290	0
D00590	1.7	0.35	145	0
D00591	4.19	0.63	23	0
D00592	3.96	0.31	135	2
D00593	3.18	0.36	45	3
D00594	1.41	0.75	54	2
D00595	2.55	1.04	189	0
D00596	1.64	0.59	153	0
D00597	2.35	1.6	231	0
D00598	2.04	0.78	108	0
D00599	2.28	0.3	59	4
D00600	3.58	0.51	274	6
D00601	2.66	0.31	350	4
D00602	1.89	0.73	77	4
D00603	3.4	0.37	159	4
D00604	3.02	0.37	67	0
D00605	1.6	0.75	41	14
D00606	1.54	0.33	220	2
D00607	4.92	0.58	158	6
D00608	4.22	0.35	593	2
D00609	2.2	0.45	41	6
D00610	2.07	0.46	102	5
D00611	2.11	0.71	52	2
D00612	2.39	0.61	192	4
D00613	5.24	0.94	8	0
D00614	2.89	1.17	245	0
D00615	3.72	1.48	339	0
D00616	3.93	0.51	180	0
D00617	1.38	0.69	99	11
D00618	5.68	0.85	248	0
D00619	6.39	1.31	10	0
D00620	1.6	0.74	53	18
D00621	1.15	0.45	67	20
D00622	4.92	0.74	7	2
D00623	3.02	1.15	320	0
D00624	2.73	0.45	342	0
D00625	1.35	0.51	65	0
D00626	6.46	0.53	3	2
D00627	1.93	0.41	131	2
D00628	7.8	0.77	10	0
D00629	1.11	0.5	22	3
D00630	2.96	0.37	40	0
D00631	5.68	0.66	259	0
D00632	7.96	1.06	275	0
D00633	2.1	1.15	131	0
D00634	3.12	1	222	0
D00635	3.56	0.42	129	2
D00636	4.14	0.9	238	0
D00637	3.16	0.91	325	0
D00638	4.64	0.55	131	1
D00639	3.49	1.24	266	0
D00640	1.03	0.46	36	0
D00641	3.53	0.52	126	5
D00642	4.22	0.63	64	4
D00643	1.28	0.36	39	4
D00644	4.2	1.01	389	7
D00645	6	1.52	997	0
D00646	3.93	1.84	249	0

D00647	2.63	0.89	321	3
D00648	3.69	0.82	377	2
D00649	1.99	0.56	120	0
D00650	3.33	0.48	86	8
D00651	3.24	0.92	484	0
D00652	1.8	0.79	119	0
D00653	1.57	0.59	250	0
D00654	2.11	0.44	141	3
D00655	2.58	0.45	120	3
D00656	3	1.55	318	0
D00657	3.62	0.94	131	2
D00658	0.9	0.47	91	6
D00659	5.1	1.48	319	0
D00660	1.55	0.49	88	2
D00661	4.4	1.14	488	0
D00662	3.8	1.41	245	0
D00663	2.51	1.11	452	0
D00664	3.15	0.95	160	2
D00665	5.03	0.99	334	0
D00666	2.35	0.46	56	10
D00667	1	0.88	95	0
D00668	3.65	0.33	384	0
D00669	3.72	0.74	308	0
D00670	2.01	0.6	30	0
D00671	1.84	0.73	112	0
D00672	3.65	1.41	212	0
D00673	3.57	1.05	160	0
D00674	3.4	0.58	190	2
D00675	2.57	1.16	154	0
D00676	2.71	0.52	214	4
D00677	2.08	0.96	123	4
D00678	3.98	0.73	97	6
D00679	2.8	0.98	247	0
D00680	2.19	0.72	98	0
D00681	1.21	0.35	69	4
D00682	2.04	0.5	68	4
D00683	3.31	1.03	154	6
D00684	3.13	1.08	320	0
D00685	1.47	0.35	83	2
D00686	1.51	0.41	24	8
D00687	4.13	0.39	145	0
D00688	1.43	0.6	49	4
D00689	2.49	0.61	73	2
D00690	2.87	0.76	286	0
D00691	3.02	1.17	198	0
D00692	2.1	0.66	79	0
D00693	1.8	0.51	117	5
D00694	5.1	0.3	369	0
D00695	1	0.36	118	0
D00696	2.92	0.72	130	2
D00697	2	0.98	242	0
D00698	3.77	0.44	149	0
D00699	6.39	0.83	237	0
D00700	3.37	1.89	125	0
D00701	4.59	0.86	285	0
D00702	1.34	0.63	54	0
D00703	2.75	0.41	128	0
D00704	3.73	0.49	89	2
D00705	2.41	0.37	168	2

D00706	3.03	0.73	126	4
D00707	3.31	1.03	110	0
D00708	1.61	0.45	71	0
D00709	4.96	0.67	28	0
D00710	2.87	1.37	242	0
D00711	4.17	0.56	187	8
D00712	7.08	0.6	548	0
D00713	3.28	0.96	126	0
D00714	1.94	0.48	148	0
D00715	3.37	1.23	215	0
D00716	1.45	0.45	209	3
D00717	4.48	0.52	152	2
D00718	3.25	1.19	205	0
D00719	2.43	0.79	137	0
D00720	1.1	0.35	85	0
D00721	4.12	0.26	68	3
D00722	2.84	1.25	178	0
D00723	1.67	0.62	112	0
D00724	3.85	1.4	588	0
D00725	4.53	0.4	316	5
D00726	4.07	1.13	349	0
D00727	4.42	0.56	102	0
D00728	2.58	0.75	163	2
D00729	4.07	0.48	11	0
D00730	3.81	0.93	222	0
D00731	1.86	0.35	211	6
D00732	1.34	0.38	59	2
D00733	1.57	0.28	91	5
D00734	2.37	0.45	52	4
D00735	5.9	1.03	391	0
D00736	2.89	1.09	39	0
D00737	2.01	0.59	91	3
D00738	3.28	0.63	105	2
D00739	2.74	0.92	191	0
D00740	2.47	0.56	217	4
D00741	2.61	0.92	131	0
D00742	5.59	1.37	295	0
D00743	1.66	0.8	110	2
D00744	5.16	0.68	287	4
D00745	3.15	0.88	78	2
D00746	5.88	0.47	77	2
D00747	2.62	0.36	137	2
D00748	1.96	0.55	154	2
D00749	1.96	0.67	21	10
D00750	1.8	0.53	78	6
D00751	4.74	0.45	73	7
D00752	2.45	0.82	97	4
D00753	2.89	1.1	125	0
D00754	2.89	0.85	188	2
D00755	4.45	0.85	94	0
D00756	3.3	0.75	297	0
D00757	1.92	0.67	202	0
D00758	4.95	0.39	237	2
D00759	4.69	0.75	159	6
D00760	6.95	0.59	248	5
D00761	4.1	1.2	96	0
D00762	3.22	0.78	40	2
D00763	5.54	1.04	163	0
D00764	3.03	0.51	233	12

D00765	2.16	0.73	191	0
D00766	3.4	0.46	186	2
D00767	2.49	1.14	377	0
D00768	2.73	1.17	79	4
D00769	9.11	0.56	57	2
D00770	6.6	0.51	321	0
D00771	4.71	0.89	147	0
D00772	5.58	1.16	167	4
D00773	2.65	1.06	72	0
D00774	2.47	0.82	273	0
D00775	3.98	0.88	220	0
D00776	3.3	0.25	122	4
D00777	5.92	0.41	130	2
D00778	3.56	0.92	229	0
D00779	2.61	1.31	264	0
D00780	2.7	1.01	338	0
D00781	3.9	0.88	71	8
D00782	2.17	1.04	203	0
D00783	2.65	1.03	84	2
D00784	2.52	1.16	142	0
D00785	2.28	0.62	92	2
D00786	3	0.99	117	4
D00787	3.29	0.8	256	0
D00788	2.75	0.53	322	0
D00789	2.21	0.68	93	0
D00790	1.48	0.4	109	3
D00791	1.64	0.44	34	6
D00792	1.93	0.5	101	2
D00793	3.53	0.56	47	3
D00794	3.82	1.31	157	0
D00795	1.93	0.4	68	0
D00796	8.39	0.47	100	4
D00797	2.47	0.98	127	0
D00798	3.31	1.12	319	0
D00799	2.71	0.97	175	0
D00800	2.59	0.5	42	0
D00801	3.31	1.15	224	0
D00802	3.13	0.86	155	3
D00803	2.44	0.78	226	4
D00804	4.06	1.39	255	0
D00805	2.24	0.41	79	0
D00806	3.1	0.63	268	2
D00807	1.92	0.74	66	5
D00808	7.31	1.11	377	3
D00809	3.35	1.16	266	0
D00810	1.69	0.54	127	10
D00811	4.8	0.48	107	8
D00812	3.39	0.94	100	0
D00813	2.4	0.47	117	3
D00814	1.97	0.52	86	3
D00815	3.67	0.28	137	4
D00816	5.96	0.6	314	0
D00817	3.98	1.14	272	0
D00818	2.99	0.85	277	0
D00819	3.15	1.11	150	0
D00820	2.36	0.92	107	0
D00821	1.39	0.53	148	0
D00822	2.95	0.42	330	5
D00823	1.98	0.63	91	2

D00824	2.07	0.36	185	0
D00825	0.8	0.28	136	0
D00826	1.86	0.55	142	2
D00827	1.87	0.6	172	0
D00828	1.59	0.4	123	1
D00829	5.4	0.74	292	0
D00830	2.25	0.49	89	2
D00831	1.86	0.41	145	0
D00832	1.32	0.34	104	2
D00833	2.48	0.75	194	0
D00834	1.91	0.64	114	4
D00835	1.16	0.41	46	0
D00836	1.8	0.6	123	2
D00837	7.5	0.44	52	2
D00838	1.96	0.33	95	2
D00839	2.55	0.58	211	6
D00840	3.3	0.41	97	0
D00841	2.59	0.46	283	1
D00842	2.19	0.93	63	0
D00843	4.26	0.89	227	0
D00844	1.73	0.52	116	4
D00845	4.43	1.12	273	2
D00846	4.19	0.31	310	4
D00847	2.4	0.92	51	0
D00848	2.01	1.27	92	2
D00849	2.32	0.7	128	0
D00850	2.55	0.77	186	0
D00851	2.4	0.65	131	1
D00852	5.34	0.69	92	8
D00853	3.41	0.74	694	1
D00854	3.76	0.57	8	0
D00855	6.63	0.41	184	3
D00856	1.13	0.3	210	5
D00857	2.16	0.82	167	2
D00858	4.9	0.54	206	4
D00859	7.35	0.9	331	0
D00860	5.06	0.94	155	1
D00861	2.24	0.95	145	0
D00862	1.83	0.31	148	2
D00863	2.75	1.11	239	0
D00864	2.02	0.57	144	0
D00865	6.2	0.42	159	0
D00866	5.42	1.21	214	0
D00867	3	0.38	191	3
D00868	3.6	2.33	290	0
D00869	0.81	0.18	103	2
D00870	2.16	0.5	259	0
D00871	2.41	0.52	273	0
D00872	2.89	1.07	210	0
D00873	1.88	0.8	125	0
D00874	3.71	0.83	600	4
D00875	7.76	1.19	95	0
D00876	4.62	0.72	406	2
D00877	2.79	0.59	158	5
D00878	5.76	0.57	310	4
D00879	2.31	1.04	3	0
D00880	1.51	1.17	107	0
D00881	8.75	0.62	560	0
D00882	2.16	0.85	164	4

D00883	1.88	0.51	42	0
D00884	1.59	0.44	114	8
D00885	2.47	0.68	124	0
D00886	2.3	0.53	101	2
D00887	3.46	0.54	230	0
D00888	3.03	0.6	104	2
D00889	2.33	0.37	133	2
D00890	5.08	0.63	229	2
D00891	3.64	1.23	68	0
D00892	4.63	0.31	154	3
D00893	2.46	0.72	225	0
D00894	6.01	1.14	154	0
D00895	2.06	0.45	147	0
D00896	2.64	0.98	174	0
D00897	1.98	0.43	87	6
D00898	5.3	0.47	280	3
D00899	3.17	1.02	190	0
D00900	3.68	1.36	134	0
D00901	1.62	0.65	166	0
D00902	3.18	0.61	277	1
D00903	1.73	0.5	105	0
D00904	6.48	0.3	188	0
D00905	0.5	0.55	71	0
D00906	2.47	0.44	215	6
D00907	3.26	1	81	4
D00908	2.11	0.64	160	5
D00909	2.44	0.7	146	0
D00910	2.36	1.43	53	0
D00911	3.19	1.5	56	2
D00912	1.87	0.55	10	12
D00913	1.24	0.4	100	0
D00914	2.58	0.67	21	0
D00915	2.4	0.64	217	2
D00916	2.16	0.72	134	0
D00917	1.58	0.53	66	10
D00918	6.93	0.55	29	2
D00919	3.66	0.79	130	0
D00920	8.24	0.78	281	0
D00921	2.62	0.95	294	0
D00922	3.33	1.44	307	0
D00923	3.49	0.59	314	0
D00924	2.85	0.63	121	0
D00925	2.45	0.62	228	2
D00926	3.28	1.49	224	0
D00927	5.83	0.52	169	0
D00928	1.67	0.76	71	0
D00929	1.3	0.29	71	4
D00930	2.08	0.49	76	8
D00931	3.1	1.14	236	0
D00932	8.11	0.94	296	0
D00933	2.26	0.93	260	0
D00934	2.29	0.58	460	4
D00935	4.06	1.16	122	4
D00936	5.13	0.6	118	4
D00937	1.9	0.38	99	2
D00938	2.97	0.74	171	0
D00939	2.4	0.55	53	2
D00940	1.9	0.57	58	10
D00941	3.57	0.63	379	3

D00942	1.9	0.66	77	14
D00943	3.06	1.14	125	0
D00944	2.2	0.47	84	5
D00945	3.39	0.24	135	1
D00946	3.32	0.67	326	4
D00947	1.92	0.5	107	0
D00948	2.38	0.81	237	0
D00949	4.21	0.41	207	0
D00950	4.54	0.56	217	2
D00951	2.52	0.43	207	3
D00952	2.78	1.16	145	0
D00953	4.6	0.77	312	4
D00954	1.57	0.46	33	10
D00955	1.98	1.22	163	1
D00956	3.85	0.78	125	0
D00957	2.79	0.99	191	0
D00958	2.39	0.67	119	2
D00959	1.55	0.57	147	0
D00960	2.16	0.46	99	2
D00961	2.43	0.57	135	0
D00962	2.67	0.72	200	0
D00963	2.6	0.99	126	0
D00964	2.53	0.57	123	1
D00965	2.04	0.64	75	3
D00966	3.53	0.79	159	0
D00967	2.74	0.74	119	2
D00968	12.23	0.73	170	0
D00969	2.26	0.79	104	2
D00970	3.27	0.75	73	4
D00971	3.52	1.01	160	2
D00972	1.8	0.58	88	4
D00973	2.48	0.66	80	6
D00974	2.51	0.65	251	2
D00975	2.85	1.25	317	0
D00976	1.86	0.29	150	0
D00977	1.51	0.46	109	7
D00978	3.14	0.62	39	0
D00979	2.07	0.97	35	8
D00980	3.74	0.91	274	0
D00981	3.79	1.08	262	0
D00982	5.49	0.61	93	2
D00983	1.54	0.53	107	6
D00984	4	1.01	238	0
D00985	2.74	0.91	139	0
D00986	5.1	0.96	140	0
D00987	0.1	0.26	120	4
D00988	4.9	1.23	207	0
D00989	3.93	1.11	182	0
D00990	2.93	0.84	186	0
D00991	2.48	0.19	70	4
D00992	3.47	1.1	270	0
D00993	2.52	0.79	51	10
D00994	2.06	0.64	174	0
D00995	2.92	1	253	0
D00996	2.94	1.11	170	0
D00997	2.12	0.79	169	0
D00998	1.84	0.51	221	0
D00999	2.96	0.34	38	2
D01000	3.27	1.14	498	0

D01001	2.1	0.85	230	0
D01002	2.41	0.67	148	0
D01003	4.1	0.44	269	2
D01004	4.48	0.74	36	6
D01005	5.3	0.33	316	6
D01006	2.51	0.83	127	6
D01007	3.67	1.23	278	0
D01008	1.95	0.93	233	0
D01009	2.54	0.27	32	3
D01010	3.48	0.98	260	0
D01011	1.54	0.53	124	4
D01012	3.8	0.66	29	2
D01013	2.56	0.66	84	0
D01014	2.03	0.38	134	6
D01015	1.44	0.49	80	4
D01016	3.63	0.93	263	6
D01017	3.85	0.51	177	4
D01018	1.77	0.58	141	4
D01019	3.7	1.18	245	0
D01020	2.5	0.83	121	0
D01021	4.75	0.96	158	0
D01022	2.23	0.6	126	0
D01023	3.87	1.26	203	0
